# A randomised, controlled study of small intestinal motility in patients treated with sacral nerve stimulation for irritable bowel syndrome

**DOI:** 10.1186/1471-230X-14-111

**Published:** 2014-06-25

**Authors:** Janne Fassov, Lilli Lundby, Jonas Worsøe, Steen Buntzen, Søren Laurberg, Klaus Krogh

**Affiliations:** 1 Department of Surgery, P, Aarhus University Hospital, Aarhus, Denmark; 2 Department of Hepatology and Gastroenterology, Neurogastroenterology Unit, Aarhus University Hospital, Aarhus, Denmark

**Keywords:** Irritable bowel syndrome, Motility tracking system-1, Small intestinal motility, Bowel habits

## Abstract

**Background:**

Irritable bowel syndrome (IBS) is among the most common gastrointestinal disorders worldwide. In selected patients with severe diarrhoea-predominant or mixed IBS subtypes sacral nerve stimulation (SNS) alleviates IBS-specific symptoms and improves quality of life. The mode of action, however, remains unknown. The present study aimed to evaluate the effect of SNS on small intestinal motility in IBS patients.

**Methods:**

Twenty patients treated with SNS for severe diarrhoea-predominant or mixed IBS were included in a randomised, controlled, crossover study. The neurostimulator was turned ON or OFF for the first one month and then to the opposite setting for the next month. Gastrointestinal transit patterns were investigated with the Motility Tracking System-1 (MTS-1) at the end of each the ON and OFF period. Primary endpoint was change in the velocity of the magnetic pill within the small intestine. Statistical testing was performed with Wilcoxon’s rank sum test and Fisher’s exact test.

**Results:**

The median velocity of the magnetic pill through the small intestine in the fasting state was not significantly different between periods with and without SNS (Group ON-OFF: median change 0 m/h (range -1.07, 0.63), Group OFF-ON: median change 0.27 m/h (range -0.59, 1.12)) (p = 0.25). Neither, was the median velocity of the magnetic pill through the small intestine in the postprandial state significantly different between periods with and without SNS (Group ON-OFF: median change -0.13 m/h (range -0.46, 0.23), Group OFF-ON: median change 0.015 m/h (range -0.48, 0.59)) (p = 0.14).

**Conclusion:**

Even though SNS may reduce symptoms of diarrhoea-predominant and mixed IBS, it has no detectable effect on small intestinal transit patterns.

**Trial registration:**

Clinical.trials.gov, (NCT00919672).

## Background

Irritable bowel syndrome (IBS) is among the most common gastrointestinal disorders worldwide. Depending on the criteria used, the reported prevalence ranges from 3-22% of the general population [1-3]. Characteristics of IBS include chronic recurrent abdominal pain associated with a change in stool form and frequency as well as relief of the abdominal pain by defecation. The aetiology of the disorder is unknown, and there are no objective markers available. Thus, diagnosis of IBS is based upon the Rome III criteria [4]. Treatment of IBS is often unsatisfactory and treatment modalities with acceptable long-term results are needed.

Sacral nerves stimulation (SNS) is a minimally invasive procedure introduced in 1995 by Matzel et al. [5]. An electrode is placed through the sacral foramen and subsequently connected with a neurostimulator to deliver continuous stimulation to the nerve fibres. Initially, SNS was used to treat idiopathic faecal incontinence. However, indications have now spread to include faecal incontinence secondary to anal sphincter lesions and severe cases of intractable constipation. A pilot study has indicated that temporary sacral nerve stimulation can reduce symptoms of IBS [6]. Recently, a randomised, controlled study from our group has shown that permanent SNS for severe diarrhoea-predominant or mixed IBS subtypes significantly alleviates IBS-specific symptoms and improves quality of life. Furthermore, SNS significantly reduces the frequency of defecation, episodes of urgency, and time spent on toilet [7].

The mechanism of action of SNS is unclear. Besides, a direct effect on the efferent sacral nerves, SNS seems to involve modulation of afferent signalling to the sacral spinal cord resulting in neuromodulation at spinal and/or supraspinal levels [8-10]. It is increasingly evident that the effects of SNS extend beyond the segments of the colorectum innervated by the sacral roots. Thus, changes in contractility and transport have been demonstrated in the right side of the colon during SNS for either faecal incontinence or severe constipation [11,12].

Studies on small intestinal motility in IBS are not in full agreement. Some have found specific abnormal motility patterns correlating to the different IBS subtypes [13,14], while others have found virtually no signs of dysmotility [15,16].

The aim of this study was to evaluate the effect of SNS on small intestinal motility in IBS patients of diarrhoea-predominant and mixed subgroups. Our a priori hypothesis was that SNS would prolong small intestinal transit.

## Methods

### Patients

**Figure 1:**
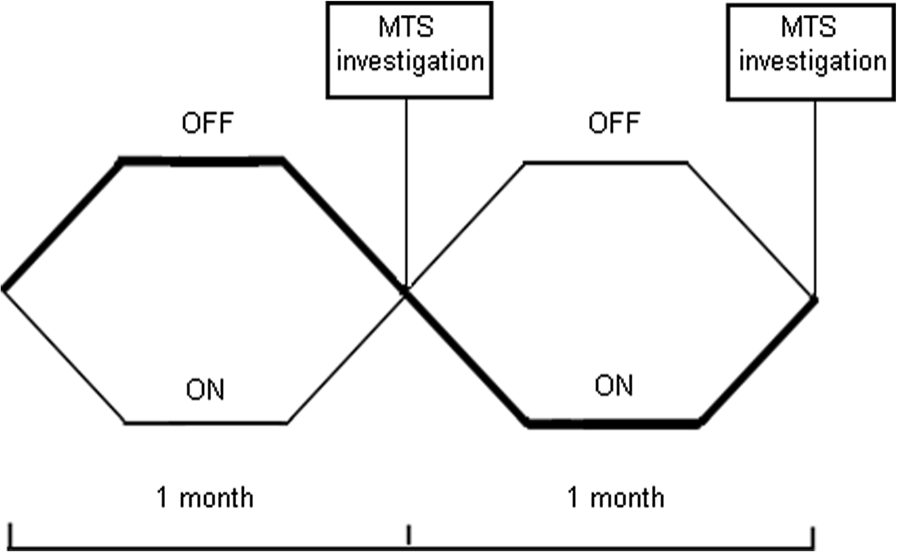
**Design of the randomised,controlled,crossover study.**

Twenty patients (5 male, median age 31 years (range 19–48)) were included in a randomised, controlled, crossover design at our tertiary centre to assess small intestinal motility after four weeks of each SNS (ON) and placebo (OFF) (Figure 1). The patients included were identical to the patients enrolled in the simultaneous study on the treatment effect of SNS for IBS that has been described in detail [7]. All patients had been diagnosed with IBS according to the Rome III criteria and were characterised as having either diarrhoea-predominant (loose (mushy) or watery stools > 25% and hard or lumpy stools < 25% of bowel movements) (n = 11) or mixed IBS (hard or lumpy stools > 25% and loose (mushy) or watery stools >25% of bowel movements) (n = 9) [17]. Patients were bloc randomised into two groups equal in size and allocation was performed by a research nurse independent of the study. Investigators were blinded to the setting of the stimulator and patients were at no time informed about the setting. Median time since implantation of the permanent neurostimulator was 4 months (range 1–20 months).

None of the patients were taking any medication affecting gastrointestinal motility. Before enrolment all patients had to present with a normal sigmoidoscopy or colonoscopy including biopsies. Furthermore, tests for celiac disease, thyroid disease, and lactose intolerance all had to be normal. Age below 18 or above 70 years, pregnancy, and severe psychological comorbidity were exclusion criteria.

Standard anal physiology tests including resting anal pressure, squeeze pressure, rectal volume tolerability, anal sensitivity, pudendal nerve motor latency, and endoanal ultrasound were performed at baseline and results were within the normal range previously published from our unit [18].

Ethical approval (The National Committee on Health Research Ethics, ID 20070218) was obtained at forehand and all patients had signed written informed consent before enrolment. The study was registered at clinical.trails.gov (NCT00919672).

### Motility Tracking System, MTS-1

This novel system tracks an orally ingested, cylindrical, silicon-covered magnetic pill (dimensions 6x15 mm, weight 0.9 g, and density 1.8 g cm^-3)^ by a 4x4 matrix of sensors positioned over the abdomen [19]. The system has previously been validated and described in detail [20].

Prior to recordings, the sensor matrix was calibrated by offsetting the earth’s and environmental magnetic fields. With the magnet ingested, the position of the sensor plate was registered with respect to anatomical reference points. During recording, the magnetic induction measured by each sensor was continuously transmitted to a computer with a sampling rate of 10 Hz. With this information, the magnets position and orientation was described according to three directions (x, y, and z) and two inclination angles (θ, φ). Changes in the magnets position coordinates reflect propagation of the magnet, while a change in the magnets orientation reflect rotation. The latter detects the specific contraction frequency characteristic of the stomach, small intestine and colon. Data processing and analysis were continuously performed running custom-made software (MTS_Record, Motilis, Lausanne, Switzerland) on a computer showing the magnets real-time position and orientation. Artefacts due to respiration and movements were detected using accelerometers placed on the neck and upper abdomen.

After an overnight fast, the magnet was ingested at 10 AM and recordings proceeded until 16 PM. A standardised meal (a sandwich and a smoothie beverage, ~ 1,500 kJ, 16% protein, 32% fat and 52% carbohydrate) was served at 14 PM.

During investigations, patients were placed in a non-magnetic bed with a head elevation (>45 degrees). They were encouraged to keep talk and movement to a minimum. Recordings were interrupted for small breaks upon request.

### Data analysis

**Figure 2:**
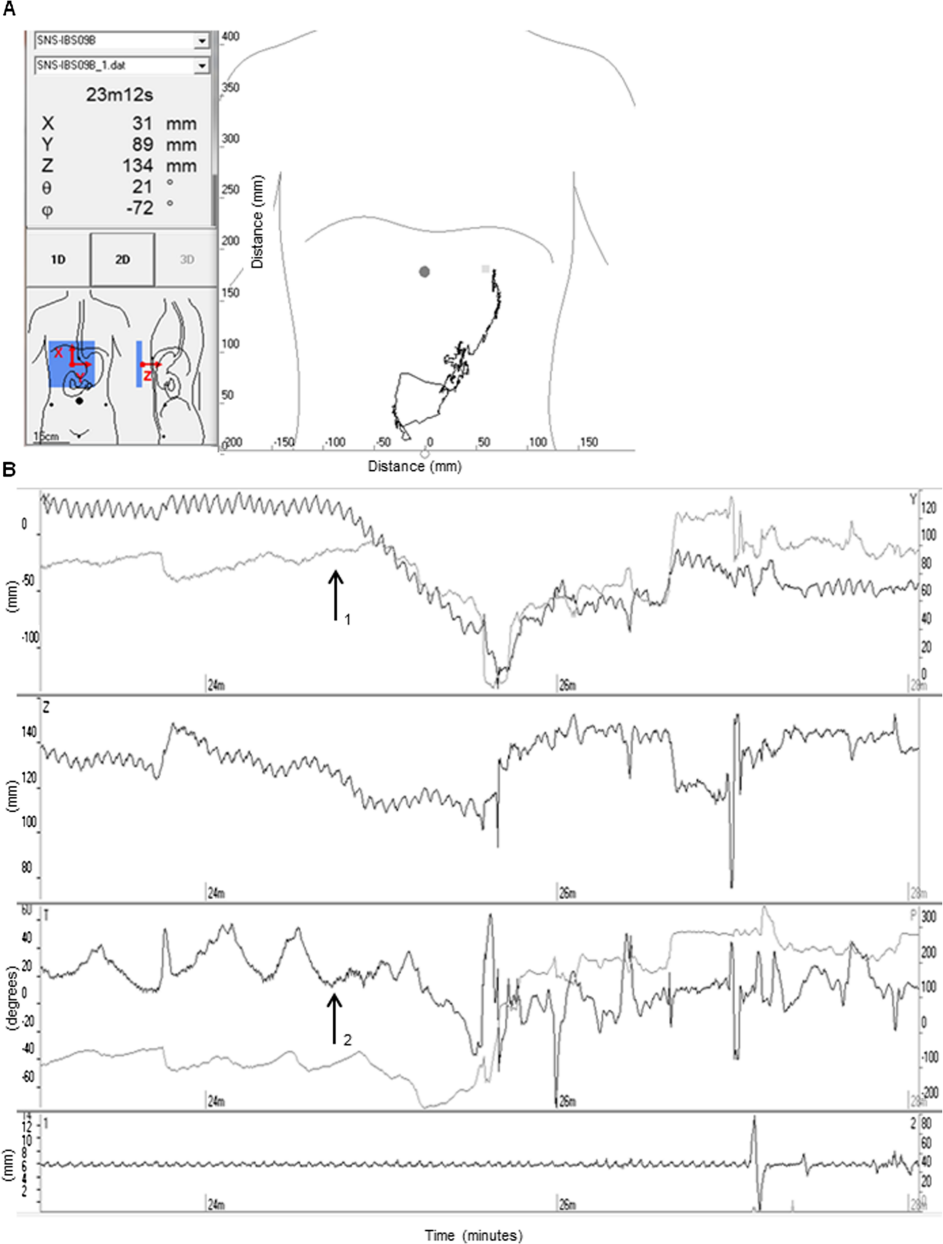
**Real time recording with MTS-1.A** In the display to the left the postion x, y, and z and the orientation θ, and φ are visualised along with the position of the sensor array over the body. To the right the concurrent recording of the magnetic pills movement through the duodenal arch is displayed. **B** The duodenal passage is visualised as the magnetic pills change in position (x, y, and z) (arrow 1) in combination with disappearance of the characteristic 3 contractions per minute patterns of the stomach (θ and φ) (arrow 2). The curve at the bottom detects artefacts from respiration and movement.

Gastric emptying was defined as the time from ingestion of the magnet until pyloric passage. Cessation of the characteristic 3 gastric contractions per minute pattern, appearance of the duodenal arch on the 2D picture, and a consecutively beginning of the characteristic 8–10 small intestinal contractions per minute pattern, were marks of pyloric passage (Figure 2). Small intestinal transit was defined as the time from pyloric passage until ileocecal passage. Cessation of the 8–10 contractions per minute pattern characteristic for the small intestine, a short burst of a fast movement, and visualisation of the magnet in the lower right quadrant of the abdomen on the 2D picture, were marks of ileocecal passage.

Dedicated software (MTS_Tool, Motilis, Lausanne, Switzerland) was used to compute the velocity (m/h) of the magnetic pill for one hour following pyloric passage and for one hour following ingestion of the standardised meal. Based on previously performed analysis of velocity histograms identifying a trimodal distribution, movements were divided into fast (>15 cm per minute), slow (between 1.5 and 15 cm per minute), and very slow (<1.5 cm per minute) [21].

### Statistics

Primary outcome parameter was the velocity of the magnetic pill within the small intestine.

The treatment effect (ON – OFF) was assessed by computing the difference between period 1 and period 2 and comparing the distribution of these differences in the two arms of the study. Likewise, the treatment period interaction was assessed by computing the sum in period 1 and period 2 and comparing the distribution of these differences in the two arms of the study.

Data are provided as median (range) and counts (percentage). Statistical analysis was performed using Wilcoxon’s rank sum test and Fisher’s exact test, with significance levels set at 0.05.

Patients included in the present study were the same as those included in a previous study on the effect of SNS on IBS specific symptoms [7]. Sample size calculation was based on expected change in symptoms and has been presented previously. Therefore, no formal sample size calculation was performed for changes in small intestinal transit time.

## Results

Patient demographics are shown in Table 1. MTS-1 investigations were well tolerated by all patients and no procedure related adverse effects were observed.

**Table 1:** Patients demographics

**Study group N = 20**	
Age (years, median range)	31(19–48)
Men/women	5/15
Body Mass Index (kg/m^2^, median range)	25.5(18–37)
Diarrhoea-predominant/mixed IBS	11/9
Duration of IBS (patients)	
1-5 years	5
6-10 years	9
> 11 years	6
Baseline symptom score in the GSRS-IBS questionnaire (median range)	62(45–80)
Baseline quality of life score in the IBS-IS questionnaire (median range)	136(82–180)

GSRS*-*IBS stands for Gastrointestinal Symptom Rating Scale – Irritable Bowel Version (13 = no symptoms of IBS to 91 = severe IBS). IBS*-*IS stands for Irritable Bowel Syndrome – Impact Scale (26 = no impact on daily life to 182 = severe impact on daily life).

### Gastric emptying

All twenty patients were eligible for comparison of gastric emptying. The characteristic 3 per minute contractions of the stomach were present in all patients both in the ON and OFF periods. We observed no statistical significant difference in gastric emptying between periods with and without neurostimulation (Group ON-OFF: median change 3.5 min (range -304, 79), Group OFF-ON: median change -33 min (range -128, 70)) (p > 0.09). No treatment period interaction was observed (p > 0.05). Gastric emptying times in the ON and OFF period are provided in Table 2.

**Table 2:** **Gastric emptying and one**-**hour velocities in the small intestine in the ON and OFF period**

	**ON period**	**OFF period**
Gastric emptying (minutes) (n = 20)	43 (5–360)	77 (4–142)
IBS-D (n = 11)	51 (9–159)	89 (34–142)
IBS-M (n = 9)	42 (5–360)	66 (4–121)
One-hour velocity following duodenal passage (meters/hour) (n = 20)	1.29 (0.56-2.3)	1.19 (0.58-1.72)
IBS-D (n = 11)	1.29 (0.67-2.9)	1.19 (0.58-1.72)
IBS-M (n = 9)	1.1 (0.56-2.3)	1.12 (0.62-1.29)
One-hour velocity postprandial (meters/hour) (n = 20)	0.48 (0.1-1.02)	0.42 (0.11-0.87)
IBS-D (n = 11)	0.49 (0.17-1.02)	0.36 (0.11-0.7)
IBS-M (n = 9)	0.48 (0.1-0.78)	0.46 (0.14-0.87)

IBS-D stand for diarrhoea-predominant IBS and IBS-M stand for mixed IBS.

Values are expressed as median and range.

### Small intestinal motility

In the fasting state, comparison of the velocity of the magnet pill during the first hour following duodenal passage was based on 19 patients. One patient had prolonged gastric emptying in the ON period (>360 minutes). The characteristic 8–10 per minute small intestinal contraction pattern was present in all patients both during ON and OFF periods. We found no statistically significant difference between the velocity of the magnet pill during periods with and without stimulation (Group ON-OFF: median change 0 m/h (range -1.07, 0.63), Group OFF-ON: median change 0.27 m/h (range -0.59, 1.12)) (p = 0.25). The same was true for both subgroups (diarrhoea-predominant and mixed IBS) (Table 3). No treatment period interaction was observed in any of the parameters (p > 0.05). Median one hour velocities in the ON and OFF period following duodenal passage are provided in Table 2.

**Table 3:** **Inter**-**period change in the velocity of the magnet**-**capsule through the small intestine**

**One-hour following duodenal passage**	**Change ON-OFF**	**Change OFF-ON**	**p**
IBS-D and IBS-M (n = 19)	0 (-1.07, 0.63)	0.27 (-0.59, 1.12)	0.25
IBS-D (n = 11)	0.09 (-0.55, 0.45)	0.14 (-0.59, 1.12)	0.68
IBS-M (n = 8)	-0.08 (-1.07, 0.63)	0.27 (0.22, 0.32)	0.32
**One-hour following ingestion of meal**	**Change ON-OFF**	**Change OFF-ON**	**p**
IBS-D and IBS-M (n = 17)	-0.13 (-0.46, 0.23)	0.01 (-0.48, 0.59)	0.14
IBS-D (n = 10)	-0.04 (-0.17, 0.09)	0.16 (-0.09, 0.59)	0.30
IBS-M (n = 7)	-0.13 (-0.46, 0.23)	-0.31 (-0.48, -0.15)	0.26

IBS-D and IBS-M stand for diarrhoea-predominant and mixed irritable bowel syndrome. Values are expressed in meters pr. hour as median change (the difference in the velocity of the magnet-capsule from ON to OFF or OFF to ON) and range.

In the postprandial state, comparison of the velocity of the magnetic pill one hour following the standardised meal was based on 17 patients. One patient had prolonged gastric emptying time and in two patients the magnet passed into the coecum, before the meal or before the end of the first postprandial hour. We found no statistically significant difference between the velocity of the magnet pill during periods with and without stimulation (Group ON-OFF: median change -0.13 m/h (range -0.46, 0.23), Group OFF-ON: median change 0.015 m/h (range -0.48, 0.59)) (p = 0.14). This was also true for both subgroups (diarrhoea-predominant and mixed IBS) (Table 3). No treatment period interaction was observed in any of the parameters (p > 0.05). Median one hour velocities in the ON and OFF period following the standardised meal are provided in Table 2.

Small intestinal passage occurred mainly during very fast movements (>15 cm pr. min) accounting only for a small proportion of time recorded. Comparing the distribution of the differences in the two arms of the study in the fasting state, there was no difference in neither the proportion of time nor the distance covered with fast, slow or very slow movements (Table 4).

**Table 4:** Small intestinal transit patterns during fast

	**ON-OFF group**	**OFF-ON group**	**p**
Proportion of time with *fast movements* (percentage)	-8 (-27, 53)	20.5 (-25, 55)	0.18
Distance covered during *fast movements* (cm)	0 (-7, 5)	2 (-5, 6)	0.09
Proportion of time with *slow movements* (percentage)	-2 (-53, 40)	-16 (-60, 35)	0.44
Distance covered during *slow movements* (cm)	-3 (-31, 10)	2.5 (-13, 22)	0.18
Proportion of time with *very slow movements* (percentage)	5 (-93, 12)	1 (-13, 7)	0.08
Distance covered during *very slow movements* (cm)	5 (-12, 32)	-5 (-23, 16)	0.06

Most of the distance covered by the magnetic pill during the first hour after pyloric passage occurred during short bursts of fast movement only accounting for a small proportion of the time. This was unaffected by SNS.

Fast movements (>15 cm/min), slow movements (between 1.5-15 cm/min), and very slow movements (<1.5 cm/min). Values are expressed as median change and range.

Within the six hours protocol the magnet pill passed into coecum in four (20%) patients during the ON period and in five (25%) during the OFF period (p = 1.00).

## Discussion

In this randomised, controlled, crossover study among patients with severe IBS we found no effect of SNS on gastric emptying or small intestinal motility. This was in spite of significantly reduced defecation frequencies, urgency episodes, and time spent on the toilet [7]. Studies on the effects of SNS on symptoms of IBS are very sparse [6,7] and the physiological effects have not previously been investigated. The lack of effect on small intestinal transit in patients with IBS is, however, consistent with previously published data on SNS in patients with faecal incontinence [22,23]. In support, this study observed no treatment period interaction in any of the parameters analysed.

In the present study, the small intestinal transit patterns were almost identical with those previously reported for healthy subjects [20]. Small intestinal dysmotility in IBS has been a matter of discussion. In a recent study using manometry there were few signs of small intestinal dysmotility [15] and others have only found abnormal small intestinal motility in IBS patients, who also had delayed gastric emptying [16]. In contrast, studies using scintigraphy or the hydrogen breath test have shown that diarrhoea-predominant IBS patients have accelerated small intestinal transit, while constipation-predominant IBS patients have delayed small intestinal transit [13,14].

The mode of action of SNS is unclear. It is, however, most likely that SNS, in addition to the direct effect on efferent sacral nerve fibres, stimulates afferent fibres from the distal colorectum to the spinal cord. Such afferent stimulation may cause neuromodulation at spinal and/or supraspinal levels. This is supported by evidence of altered motility in the right side of the colon during SNS [11,12]. Furthermore, SNS alters cerebral evoked potentials and reduces the overall corticoanal excitability during rectal distension [9,24]. Thus, modulation through afferent nerve fibres may be an important part of the mode of action of SNS for IBS.

In the present study, SNS had a significant effect on the frequency of defecation, episodes of urgency, and time spent on toilet. Based on results from the same patient group, we have previously reported that SNS alleviates IBS specific symptoms including pain, bloating, diarrhoea, constipation and satiety and that the effect lasts at least one year [7]. In patients with faecal incontinence, the percutaneuos nerve evaluation test alters rectal sensation to distension [9,24,25]. Most IBS patients have visceral hypersensitivity [26,27] and abnormal engagement of CNS regions associated with emotional arousal and endogenous pain modulation has been documented during rectal distension [28]. It is therefore possible, but remains to be investigated, that SNS alters rectal sensitivity and central processing of stimuli in IBS patients.

The strength of the present study is the randomised design. There are, however, important limitations and the relatively small patient number may have caused a type II error.

Even though patients were never informed of the actual setting of their stimulator, fifteen out twenty patients were able to tell correctly, whether the stimulator was turned ON or OFF, wherefore, the study is not truly double-blinded [7]. This may have caused a placebo effect. On the other hand, all symptomatic effects of SNS were maintained at least one year after implantation, which speaks against the effect being solely placebo [7]. Moreover, setting the stimulation subsensory in patients with constipation has been proved to eradicate the effect on the colonic motility [29].

Time from implantation of the permanent stimulator to inclusion varied considerably among patients in the study. In a study from our unit on patients treated with SNS for faecal incontinence, symptoms reappeared within a few hours after withdrawal of stimulation [30] and in previous motility studies, the neurostimulators were turned off only for one week before investigations [22,23]. Most important, we observed no treatment period interaction in the present study or in the study evaluating the effects of SNS on IBS-specific symptoms and quality of life [7]. This speaks against that time from implantation to inclusion into the study is affecting small intestinal motility during SNS.

During fast a non-digestible object as the magnetic pill will usually leave the stomach with an antral phase III of the MMC [31,32]. This restricts the usefulness of MTS-1 for estimating gastric emptying time. However, in accordance with our results the scintigraphic study by Damgaard et al. failed to detect any effect of SNS on gastric emptying [23].

Practical and ethical reasons mandated that the recordings were limited to six hours as patients had to be almost immobile. Therefore, we were only able to define the total small intestinal transit time in a few patients. Such data could have been obtained by scintigraphy. In contrast to scintigraphy, the MTS-1 system allows continuous description of transit patterns and the MTS-1 has previously allowed identification of abnormal small intestinal motility in patients with systemic sclerosis, spinal cord injury and carcinoid syndrome [33-35]. The study protocol for the present study was based on experiences from those previous studies.

Gastric emptying is very variable and with the protocol limited to six hours, the assessment of the fasting small intestinal motility was based on measurements during the first hour after pyloric passage. This is too short to allow us to draw any conclusions about the effects of SNS on the migrating motor complex (MMC). Furthermore, the six hours protocol proved too short to allow detailed description of colorectal motility even if patients were studied on consecutive days.

## Conclusion

SNS holds promise as an effective treatment modality against severe diarrhoea-predominant or mixed IBS. Even though, SNS reduces the frequency of defecation, episodes of urgency, and time spent on toilet, this occurs without major changes in small intestinal transit patterns. We speculate that the effects of SNS in IBS may be caused by modulation of afferent nerve fibres causing altered colorectal sensory perception.

## Competing interests

L Lundby and S Buntzen have received honoraria from Medtronic Inc as speakers. S Laurberg has received an honorarium as a member of Medtronic Inc’s medical advisory board. Med Inc had no had no influence on study design, data analysis or interpretation, writing, or submission of the report. J Fassov, J Worsøe, and K Krogh all declare to have no competing interests.

## Authors’ contributions

JF, LL and KK were the writing committee. JF and JW analysed data. JF, LL, SB, SL, and KK were all involved in the design of the study, patient enrolment and follow-up, and manuscript review. All authors read and approved the final manuscript.

## Pre-publication history

The pre-publication history for this paper can be accessed here:

http://www.biomedcentral.com/1471-230X/14/111/prepub
